# A longitudinal evaluation of the impact of the COVID-19 pandemic on patients with pre-existing anxiety disorders

**DOI:** 10.1017/ipm.2021.32

**Published:** 2021-04-05

**Authors:** K. Hennigan, M. McGovern, R. Plunkett, S. Costello, C. McDonald, B. Hallahan

**Affiliations:** 1 Galway–Roscommon Mental Health Services, University Hospital Galway, Galway, Ireland; 2 School of Medicine, National University of Ireland Galway, Galway, Ireland

**Keywords:** Anxiety disorders, COVID-19, obsessive-compulsive disorder

## Abstract

**Objectives:**

To examine if the COVID-19 pandemic is associated with a differential effect over time in relation to its psychological and social impact on patients with established anxiety disorders.

**Methods:**

Semi-structured interviews were conducted with 24 individuals attending the Galway–Roscommon Mental Health Services with an International Classification of Diseases (ICD)-10 diagnosis of an anxiety disorder at two time points (six months apart) to determine the impact of the COVID-19 restrictions on anxiety and depressive symptoms, social and occupational functioning and quality of life.

**Results:**

No statistical difference in symptomatology was noted between the two time points in relation to anxiety symptoms as measured by utilising psychometric rating scales (BAI and HARS) or utilising a Likert scale. The greatest impact of COVID-19 at both time points is related to social functioning and quality of life. Significant variability was noted for individual participants. Qualitative analysis noted social isolation, concern for the participants’ future and increased difficulty managing anxiety with ongoing restrictions.

**Conclusions:**

No significant overall change in symptomatology or functioning over time was noted for individuals with pre-existing anxiety disorders. Variability was, however, demonstrated between individuals, with some individuals describing ongoing anxiety, social isolation and concern for their future. Identifying those with ongoing symptoms or distress and providing multidisciplinary support to this cohort is suggested.

## Introduction

COVID-19 is the infectious disease associated with the recently discovered coronavirus, SARS-CoV-2. First identified in Wuhan, China, in December 2019, COVID-19 was characterised as a pandemic by the World Health Organization (WHO) on 11 March 2020 (World Health Organisation, 2020). The declaration of the pandemic was followed by the implementation of restrictions and ‘lockdowns’ in many countries worldwide. In Ireland, these restrictions included ‘cocooning’ of individuals over 70 years of age, a limitation on travel (2 km radius at one point from one’s accommodation with some exceptions) and the introduction of social distancing measures, which resulted in the closure of many facilities deemed as ‘non-essential’. In addition to restaurants and cafes, these facilities included centres attended by individuals with mental health disorders such as day hospitals and day centres. From May 2020, there was a gradual easing of restrictions, however, due to increasing cases of COVID-19 in Ireland, the government on the advice of the National Public Health Emergency Team (NPHET) increased restrictions again based on a five-level system to Level 5 on 19 October 2020. These increased restrictions were similar to those initially introduced with the exception of schools and pre-school facilities remaining open, a limitation of travel from one’s residence set at 5 km and no mandated requirement for ‘cocooning’ of individuals over 70 years of age.

Whilst initial public debate focused predominantly on the physical health sequelae of contracting COVID-19 and economic consequences of COVID-19 mandated restrictions, increasing debate and discussion in both medical literature and in social media has surrounded the potential adverse psychological or psychiatric sequelae relating to COVID-19-enforced restrictions. Previous viral pandemics have been associated with increased psychological distress (WHO ‘Outbreak Communication Guidelines’, 2005), with perspective pieces (Pfefferbaum and North, 2020; Kelly, 2020) and some initial research studies noting an increase in psychiatric pathology, including higher levels of depressive and anxiety symptoms, in individuals with no prior mental disorder subsequent to mandated government restrictions secondary to COVID-19 (Wang et al., 2020). However, there is limited research to date assessing the impact of the COVID-19 pandemic on individuals with pre-existing diagnosed mental health disorders who are attending secondary mental health services. Anxiety disorders are important to explore given the potential adverse impact on the anxiety of COVID-19 (Wang et al., 2020). Obsessive-compulsive disorder (OCD) is particularly worthy of exploration given current strong recommendations on handwashing, as this anxiety disorder is marked by the presence of recurrent obsessional thoughts encompassing contamination with associated compulsive rituals incorporating repetitive handwashing and cleaning, and/or undertaking disproportionate measures to reduce exposure to perceived sources of contamination (Rasmussen and Eisen, 1992; Murphy et al., 2010). We previously noted a relatively modest impact of COVID-19 on anxiety symptoms for individuals with pre-existing anxiety disorders (Plunkett et al., 2020), with social functioning most impacted due to COVID-19 restrictions. This study, however, assessed individuals 5–7 weeks after government-mandated social restrictions had commenced. However, with restrictions ongoing since then at various levels of stringency, the impact of these ongoing mandated restrictions in this population has yet to be examined.

Consequently, in this study, we wanted to assess the psychological and social impact of COVID-19 including its associated mandated social restrictions on individuals with diagnosed anxiety disorders attending a general adult mental health service longitudinally. We hypothesised that participants would have increased symptomatology and impaired social functioning compared to initial assessments six months earlier.

## Methods

### Participants

All patients who previously engaged in a study examining the impact of the COVID-19 pandemic on patients with pre-existing anxiety disorders (*n* = 30) (Plunkett et al., 2020) were invited to participate in this study by letter and subsequently phoned to provide clarification regarding the purpose of and procedure associated with this study. Anxiety disorders as previously detailed consisted of those related to triggering events denoted as ‘trigger disorders’ and included OCD, social phobia and agoraphobia and those predominantly unrelated to a trigger event denoted as ‘non-trigger disorders’ and included GAD, panic disorder and mixed anxiety and depressive disorder, with all clinical diagnoses based on International Classification of Diseases (ICD)-10 diagnostic criteria. Inclusion criteria required the patients to have one of these listed anxiety disorders, be over 18 years of age and having capacity to provide written informed consent for study participation. Research interviews were undertaken by psychiatrists with several years of clinical practice (KH, MMcG, SC, RP, BH) with training in study procedures provided by the principal investigator (BH). All responses were anonymised and all data were stored securely and handled in accordance with the Data Protection Act, 2018. Ethical approval was attained prior to study commencement from the Galway University Hospitals Research Ethics Committee.

### Procedure

All individuals previously provided written informed consent and consent was reattained verbally for this study. For individuals providing informed consent for engagement in the follow-up study (*n* = 24, 80% response rate – one person refused and five individuals were uncontactable), clinical case notes were reviewed to ascertain if there were any changes relating to clinical data, including changes in prescribed psychotropic medications including dose of medications, where participants described uncertainty pertaining to their treatment regimen.

### Assessments

A semi-structured interview was conducted by telephone (in line with government and health service policy) between 15 October and 29 October 2020, approximately 6 months after individuals participated in baseline assessments and at this repeat assessment coincided with government-mandated social restrictions increasing to ‘Level 5’ restrictions.

Demographic and clinical variable data are additionally attained in this study related to physical health status including COVID-19 diagnosis and testing status, and the effect of COVID-19 on the participants’ employment or vocational status and/or site of employment. Categorical data pertaining to the effect of COVID-19 on participants’ mental health status overall and severity of anxiety symptoms (better, no change, worse) was attained. Participants’ subjective experience of the impact of COVID-19 pandemic was measured by utilising the same Likert scales at both time points (0–10) to measure: (1) anxiety symptoms, (2) mood symptoms (3) social functioning, (4) occupational functioning and (5) quality of life; with 0 indicating no adverse impact and 10 indicating a very severe impact due to restrictions imposed because of the COVID-19 pandemic.

The same established psychometric instruments with known high reliability and validity indices were additionally utilised at both time points to measure current symptomatology and included the: (1) Beck Anxiety Inventory (BAI, Beck & Steer, 1993), (2) Hamilton Anxiety Rating Scale (HARS, Hamilton 1959), (3) Clinical Global Impression – Severity (CGI-S, Guy 1976), (4) Global Assessment of Function (GAF, Hall 1978) and (5) the Yale–Brown Obsessive Compulsive Scale (Y-BOCS, Goodman et al. 1989) (for participants with a diagnosis of OCD only (*n* = 11)). The Clinical Global Impression – Improvement (CGI-I) scale was utilised to compare the participants’ overall mental state at the follow-up visit compared to the baseline observation. Free-text data pertaining to participants’ perspectives on the impact of COVID-19 for them were also invited.

### Statistical analysis

Statistical analysis was performed using the Statistical Package for Social Sciences (SPSS) 24.0 for Windows (SPSS Inc., IBM, USA). Descriptive analyses (frequencies, percentages, means and standard deviation) on key demographic and clinical data were performed for both categorical and continuous variables as appropriate. We utilised the paired *t*-test for parametric data to compare psychometric data between baseline and follow-up visits. Data were examined to determine if normally distributed by visual inspection utilising histograms and by Q–Q plots and non-parametric testing were additionally undertaken as appropriate, with the Wilcoxon ranked test utilised (with median and interquartile ranges also attained) to compare data between both time points. Chi Square (χ^2^) test was additionally utilised for some non-parametric data as appropriate. CGI-I data were combined due to the relatively small sample size into three categorical variables: improved (1–3), no change (4) or disimproved (5–7).

Changes in psychometric data over time were examined for the entire group with analyses repeated for participants with ‘trigger’ or ‘non-trigger’ disorders, for individuals with and without a diagnosis of OCD and for individuals with and without a diagnosis of a comorbid mental health or physical health disorder. The related sample McNemar change test was undertaken to ascertain changes in the category of anxiety symptoms as measured by the BAI or HARS. All statistical tests were two-sided and the α-level for statistical significance was 0.05.

Free-text data were examined and were open-coded based on the framework of the questionnaire and on any other themes unrelated to these questions that emerged. This data attained from free texts was then grouped into themes by consensus of the researchers (KH, SC, MMcG, BH).

## Results

### Demographic and clinical data

Of the 30 participants in the initial study, 24 (80%) were available for follow-up interviews. There was no significant difference in age, gender or diagnosis between respondents and non-respondents. Data for the 24 participants is presented in Table 1. Of note, 16 participants were female (66.7%), 4 participants lived alone (16.0%) and the mean age of participants was 37.4 (*SD* = 11.4) years. Nine individuals had been in employment prior to COVID-19, with only three individuals (33.3%) maintaining their employment at time point 1 and only one participant (11.1%) still in employment at time point 2. Three participants underwent testing for COVID-19, at some stage since the commencement of the COVID-19 pandemic, all testing negative. Fourteen (58.3%) participants fulfilled criteria for an anxiety disorder denoted as a ‘trigger anxiety’ disorder. The most common anxiety disorder was OCD (*n* = 11, 45.8%) followed by GAD (*n* = 6. 25.0%). Twelve (50%) participants fulfilled diagnostic criteria for an additional mental health disorder with an emotionally unstable personality disorder of borderline type most common (*n* = 4, 16.7%), and 5 (20.8%) participants were diagnosed with comorbid physical disorders. Twenty-one (87.5%) participants were prescribed psychotropic medication with 10 (41.7%) participants prescribed a Selective Serotonin Reuptake Inhibitor (SSRI), and 8 (33.3%) participants prescribed a Serotonin and Noradrenaline Reuptake Inhibitor (SNRI). Nine (37.5%) participants were prescribed more than one psychotropic medication.

**Table 1.** Demographic and clinical variable

Variable	*n* (%)
**Gender**	
Male	8 (33.3)
Female	16 (66.7)
**Marital status**	
Single	13 (54.2)
Married/civil partnership	5 (20.8)
Separated/divorced	6 (25.0)
**Employment status**	
Unemployment	15 (62.5)
Employed	9 (37.5)
Lost employment	8 (88.9)
**Anxiety disorder**	
‘*Trigger anxiety disorders’*	
Obsessive-compulsive disorder	11 (45.8)
Social phobia	2 (8.3)
Agoraphobia	1 (4.2)
‘*Non-trigger anxiety disorders’*	
Generalised anxiety disorder	6 (25.0)
Mixed anxiety and depression	3 (12.5)
Panic disorder	1 (4.2)
**Domiciliary status**	
Parents	7 (29.2)
Partner/spouse	7 (29.2)
Other family members	4 (16.7)
Housemates/friends	2 (8.3)
Alone	4 (16.7)
**Substance use**	
Alcohol^a^	13 (54.2)
Nicotine	3 (12.5)
Cannabis^a^	1 (4.2)
Other psychoactive substances	0 (0.0)
**Throat and nasal swab test for COVID-19**	
Yes^b^	3 (12.5)
No	21 (87.4)
**Comorbid psychiatric disorder**	
EPD of borderline type	4 (16.7)
Schizophrenia	3 (12.5)
Anorexia nervosa	3 (12.5)
Other disorders^c^	2 (8.3)
**Comorbid physical disorder**	
Diabetes mellitus	1 (4.2)
COAD/asthma	1 (4.2)
Other physical health disorders^d^	3 (12.5)

COAD, Chronic Obstructive Airway Disease; EUPD, Emotionally Unstable Personality Disorder.

a No participants fulfilled the criteria for harmful alcohol use or dependence.

b All individuals were tested negative for COVID-19.

c Includes Autism Spectrum Disorder.

d Includes neurological, inflammatory and musculoskeletal disorders.

### Change in symptomatology

As demonstrated in Table 2a, no statistical difference in symptomatology was noted between the two time points when analysing the total group in relation to anxiety symptoms as measured by utilising psychometric rating scales (BAI and HARS) or utilising a Likert scale. Similarly, there was no difference in levels of symptoms of OCD, or on measures of mood, social or occupational functioning or quality of life. The greatest impact of COVID-19 was related to social functioning at both time points followed by quality of life. As some Likert scale data were non-parametrically distributed, analysis was repeated by utilising the Wilcoxon Rank test and no difference in results was demonstrated (Table 3). When examining if individuals had switched category in relation to the category of severity of anxiety symptoms, individual variation was noted for both the BAI and HARS (Table 4), with this more evident with the BAI. Five individuals (26.3%) moved from a mild category of symptoms in the BAI to the moderate/severe symptom range, with two individuals (40.0%) moving from the moderate/severe symptom range into the mild symptom range.

**Table 2(a).** Change in psychometric scores: total group

Variable	Baseline Mean (SD)	Follow-up Mean (SD)	Difference (95% CI)	Statistics *t*, *p*
**BAI**	13.04 (13.12)	14.58 (12.92)	−1.54 (−6.16, 3.08)	0.69, 0.50
**HARS**	11.08 (7.94)	10.96 (8.47)	0.38 (−2.31, 3.06)	0.29, 0.78
**Y-BOCS**				
Obsessions	6.78 (3.70)	7.22 (4.97)	−0.44 (−2.75, 1.87)	0.44, 0.67
Compulsions	6.33 (3.32)	6.22 (3.15)	0.11 (−2.67, 2.89)	0.09, 0.93
Total	13.11 (6.39)	13.67 (6.96)	−0.56 (−2.05, 0.94)	0.86, 0.42
**GAF**	62.38 (11.72)	60.21 (17.13)	2.17 (−4.13, 8.46)	0.71, 0.48
**Likert scales**				
Anxiety	3.82 (3.06)	3.42 (2.78)	0.42 (−0.88, 1.71)	0.66, 0.52
Mood	3.38 (3.25)	2.67 (2.58)	0.71 (−0.88, 2.30)	0.92, 0.37
Social functioning	4.54 (2.94)	4.00 (2.93)	0.54 (−1.07 (2.15)	0.70, 0.49
Occupational functioning	3.04 (3.46)	2.54 (3.16)	0.50 (−0.97, 1.97)	0.71, 0.49
Quality of life	4.33 (2.63)	3.83 (2.84)	0.50 (−0.92, 1.92)	0.73, 0.47

**Table 2(b).** Change in psychometric scores: trigger disorders (n = 14)

Variable	Baseline Mean (SD)	Follow-up Mean (SD)	Difference (95% CI)	Statistics *t*, *p*
**BAI**	9.86 (10.73)	12.29 (12.32)	−2.43 (−9.77, 4.92)	0.71, 0.49
**HARS**	9.79 (6.75)	10.14 (8.63)	−0.36 (−4.47, 3.76)	0.19, 0.85
**Y-BOCS**				
Obsessions	6.78 (3.70)	7.22 (4.97)	−0.44 (−2.75, 1.87)	0.44, 0.67
Compulsions	6.33 (3.32)	6.22 (3.15)	0.11 (−2.67, 2.89)	0.09, 0.93
Total	13.11 (6.39)	13.67 (6.96)	−0.56 (−2.05, 0.94)	0.86, 0.42
**GAF**	61.21 (14.13)	56.93 (21.46)	4.29 (−6.89, 15.46)	0.83, 0.42
**Likert scales**				
Anxiety	3.36 (2.73)	2.71 (2.61)	0.64 (−0.78, 2.07)	0.97, 0.35
Mood	3.43 (3.16)	1.79 (2.01)	1.64 (−0.31, 3.60)	1.81, 0.93
Social functioning	4.43 (2.90)	3.64 (3.03)	0.79 (−1.20, 2.78)	0.85, 0.41
Occupational functioning	3.00 (3.42)	2.64 (3.56)	0.36 (−1.89, 2.61)	0.34, 0.74
Quality of life	4.07 (2.50)	3.00 (2.96)	1.07 (−0.60, 2.74)	1.39, 0.19

**Table 2(c).** Change in psychometric scores: non-trigger disorders (*n* = 10)

Variable	Baseline Mean (SD)	Follow-up Mean (SD)	Difference (95% CI)	**Statistics*****t***, ***p***
**BAI**	17.50 (15.35)	17.80 (13.71)	−0.30 (−6.26, 5.66)	0.11, 0.91
**HARS**	12.90 (9.43)	11.50 (8.40)	1.40 (−2.40, 5.20)	0.83, 0.43
**GAF**	64.00 (7.59)	64.80 (6.68)	−0.80 (−2.98, 1.38)	−0.83, 0.43)
**Likert scales**				
Anxiety	4.50 (3.50)	4.40 (2.83)	0.10 (−2.69, 2.88)	0.08, 0.94
Mood	3.30 (3.56)	3.90 (2.88)	−0.60 (−3.51, 2.30)	0.47, 0.65
Social functioning	4.70 (3.13)	4.50 (2.88)	0.20 (−2.98, 3.38)	0.14, 0.89
Occupational functioning	3.10 (3.70)	2.40 (2.67)	0.70 (−1.43, 2.83)	0.74, 0.48
Quality of life	4.70 (2.91)	5.00 (2.31)	−0.30 (−3.12, 2.52)	0.24, 0.82

CGI-I, Clinical Global Impression – Improvement; CGI-S, Clinical Global Impression – Severity; BAI, Beck Anxiety Inventory; HARS, Hamilton Anxiety Rating Scale; GAF, Global Assessment of Function; Y-BOCS, Yale–Brown Obsessive Compulsive Scale.

**Table 3.** Psychometric variable data (non-parametric analysis)

Variable	Baseline Median (IQR)	Follow-up Median (IQR)	Statistics *z*, *p*
**BAI**	9.5 (3.0–19.5)	12.5 (2.5–22.8)	0.713, 0.476
**HARS**	9.0 (5.0–16.0)	9.0 (5.0–14.0)	0.424, 0.762
**Y-BOCS**			
Obsessions	6.0 (5.0–10.0)	5.0 (4.5–11.0)	0.287, 0.744
Compulsions	6.0 (5.0–11.0)	6.0 (4.5–8.5)	0.175, 0.861
Total	13 (11.0–20.0)	11.0 (10.0–19.0)	0.689, 0.491
**GAF**	65.0 (56.0–70.0)	65.0 (56.8–67.8)	0.570, 0.585
**Likert scales**			
Anxiety	3.5 (0.5–7.0)	3.0 (0.5–5.0)	0.571, 0.568
Mood	3.0 (0.0–6.0)	2.0 (0.1–4.8)	0.416, 0.677
Social functioning	5.0 (2.0–7.0)	5.0 (0.3–6.8)	0.716, 0.474
Occupational functioning	1.0 (0.0–6.0)	0.5 (0.0–6.0)	0.625. 0.532
Quality of life	5.0 (2.3–6.0)	4.0 (0.5–6.0)	0.683, 0.488

Wilcoxon rank test is utilised.

Data pertaining to clinical symptomatology for individuals characterised as ‘trigger’ and ‘non-trigger’ disorders are presented in Tables 2b and 2c. No difference over time was noted across psychometric instruments or Likert scales for either group. Individuals with non-trigger anxiety disorders had higher levels of subjective anxiety symptoms as measured with the BAI and anxiety Likert scale at both time points compared to those with trigger disorders.

The presence of a comorbid psychiatric or physical health disorder was not associated with a statistically significant change in symptomatology over time.

### Qualitative data

Twenty (83.3%) participants provided free-text responses. In total, four themes emerged: (1) neutral effects of COVID-19 (*n* = 6), (2) negative psychological impact of COVID-19 (*n* = 5), (3) negative social impact of COVID-19 (*n* = 5) and (4) concern for the future due to COVID-19 (*n* = 4). These comments as detailed in Box 1 highlighted that whilst many participants had no adverse sequelae secondary to the COVID-19 pandemic, the reduction in social contact, often organised or associated with mental health or other health services was a significant concern and/or a source of distress for some patients. Additionally, several patients reported increased anxiety or an increase in subjective low mood secondary to isolation or excessive engagement with media coverage of the COVID-19 pandemic. The free-text responses noting changes in symptomatology and functioning corresponded with individuals who changed category from the mild symptom range on the BAI to the moderate or severe symptom range or vice versa (see Box 1 and Table 4).

Box 1. Themes emanating from free-text responses

**Theme 1 Neutral effects of COVID-19 (** ***n*** **= 6)**

- *‘No significant effect on my life’* (#24, Female)
- *‘Easier now as restrictions are not unknown’* (#45, Female)

**Theme 2: Finding restrictions more difficult (** ***n*** **= 5)**

- ‘*I am finding* the restrictions more difficult to manage this time’ (#8, Male)
- ‘*Managing with COVID-19 is more difficult, it is worse this time, I am more anxious’* (#33, Female)

**Theme 3: Negative social impact of COVID-19** (*n* = 5)

- ‘*I cannot meet friends for a coffee as I would usually do’ (#4, Female)*
- *‘I feel lonely and isolated’ (#47, Female)*
- *‘I feel more isolated than before, I am worse now’ (#17, Female)*
- *‘I miss my work colleagues’ (#11, Male)*

**Theme 4: Concern for the future because of COVID-19 (** ***n*** **= 4)**

- ‘I am worried for my future work opportunities’ (#37, Female)
- ‘I feel fearful for the future’ (#5, Male)
- ‘It is like a constant war, with no end in sight’ (#49, Male)

Of note, #8 and #17 changed category from mild symptom range on the BAI and HARS to the moderate–severe category, with #45 changing category on the BAI and HARS from the moderate–severe range of symptoms to the mild symptom range.

**Table 4.** Psychometric instruments and caseness

	Baseline ***n*** **(%)**	**F**ollow-up ***n*****(%)**	Test statistic,^*^ *p*
**BAI**			
Mild symptoms (BAI ≤ 21)	19 (79.2)	Mild = 14 (73.7)	0.571, 0.450
		Moderate/Severe = 5 (26.3)	
Moderate/severe symptoms (BAI ≥ 22)	5 (20.8)	Mild = 2 (40.0)	
		Moderate/Severe = 3 (60.0)	
**HARS**			
Mild symptoms (HARS ≤ 17)	19 (82.6)	Mild = 17 (89.5)	<0.01, 1.00
		Moderate/Severe = 2 (10.5)	
Moderate/severe symptoms (HARS ≥ 18)	4 (17.4)	Mild = 2 (50.0)	
		Moderate/Severe = 2 (50.0)	

* Related sample McNemar change test.

## Discussion

To our knowledge, this is the first longitudinal study examining the impact of COVID-19 restriction on those with pre-existing diagnosed anxiety disorders who are attending secondary mental health services. Participants reported a deleterious impact of COVID-19 on anxiety symptoms; however, this impact was not marked, with no significant overall change in symptomatology over time.

An increase in anxiety symptoms secondary to COVID-19 has been noted in individuals without pre-existing mental disorders. For example, in a Chinese cohort, approximately 20% of individuals demonstrated anxiety and/or depressive symptoms of at least moderate severity (Li et al., 2020); compared to 4% of individuals in a study a year earlier (Huang et al., 2019). These results were replicated in a large online survey in a study conducted in the United States of America, which noted a threefold increase in anxiety and/or depressive symptoms (˜35% *v.* 11%) in April–May 2020 compared to the same time period the previous year. Of note, in this survey, no overall increase in anxiety or depressive symptoms was noted in four samples of participants since April 2020 (Twenge and Joiner, 2020). Similarly, a 4-week follow-up study in a general population cohort in China noted no further increase in anxiety or depressive symptoms with a modest reduction in overall distress associated with COVID-19 noted, however, less than 40% of individuals completed surveys at both time points (Wang et al., 2020). Thus, consistent with our research, these surveys evaluating anxiety and depressive symptoms noted no deterioration of symptoms longitudinally since the onset of COVID-19 and its associated restrictions; however, unlike our study, these studies were conducted in individuals not attending mental health services and the duration of time between repeat surveys (with most participants not being involved in prior surveys) was shorter in duration than our study. An Irish survey conducted approximately 3 weeks after initial COVID-19 restrictions commenced noted that 28% of those screened fulfilled criteria for either generalised anxiety disorder or a depressive episode (Hyland et al., 2020). Together, these findings suggest that there is an increase in anxiety and depressive symptoms, likely related to the COVID-19 pandemic, however, it is likely individuals with such symptomatology where required are attaining supports within primary care rather than secondary mental health services.

There are a number of putative reasons why to date; there has been no overall deterioration in symptomatology and functioning (which is opposite to what was hypothesised) in this study. Overall, this cohort of patients attending a community mental health team is largely stable from a mental health perspective, *albeit* with some ongoing anxiety symptoms. Significant heterogeneity was noted, however, and thus despite no statistically significant change in symptomatology for the entire cohort, there was evidence for some individuals of a change in the severity of anxiety symptoms both utilising psychometric instruments and in the free-text comments. This finding is reflective of a recent research study that noted despite no significant overall deterioration in symptomatology for their study cohort; that some individuals, particularly those with a prior history of mental disorder demonstrated deterioration in symptomatology (as measured by daily hassles) on longitudinal assessments (Ahrens *et al.*, 2021). Additionally, many participants continue to attain some ongoing supports as appropriate from a community mental health team member(s), and have an awareness of how to access supports with many participants aware of anxiety management techniques or other coping strategies as a result of engagement with mental health services. None of the individuals in this cohort are currently engaged in harmful use of psychoactive substances and although 54% of the cohort consumes alcohol, no participant engages to our knowledge in consuming alcohol above the Health Service Executive recommended low-risk alcohol guidelines (https://www2.hse.ie/wellbeing/alcohol/improve-your-health/). A diagnosis of a mental disorder does not mitigate against an individuals’ ability to be resilient (Hermann et al 2011), and it is probable that several of the study participants have adapted positively to maintain their mental health, despite the adversity experienced with COVID-19 and its associated restrictions.

Some participants, however, have displayed an increase in symptomatology, reduced functionality and reduced quality of life. Of note, 70% of the qualitative comments were negative in nature despite no statistical difference in the level of symptomatology or functioning at time point 2 compared to time point 1. Qualitative comments described increased social isolation, increased anxiety symptoms with less ability to manage anxiety with ongoing restrictions (particularly with the onset of Level 5 restrictions) and concern for the future, pertaining to future employment and longevity of enforced restrictions. Only one study participant is currently in active employment out of the nine who were engaged in employment prior to the onset of COVID-19-enforced restrictions in March 2020. The identification of individuals who have had deterioration in functionality and increased symptomatology for additional multidisciplinary support is thus merited, despite overall rates of symptomatology and functioning in the study cohort not changing to date.

There are a number of limitations with this study, the most significant of which was the modest sample size of 24 participants and the absence of a control group. However, 80% of the original cohort engaged in the follow-up study and there was no difference in socio-demographic or clinical factors between individuals who did and did not participate in this follow-up study. Additionally, caution is required in the interpretation of findings between different anxiety disorders given the relatively low sample size. This study was undertaken within one community mental health team, and thus it is possible that the findings may not be generalisable to other services with differential resources or comorbid disorders. Finally, separating individuals with anxiety disorders into ‘trigger’ and ‘non-trigger’ groups is not currently a recognised categorisation. We examined individuals with OCD alone compared to other anxiety disorders, in addition to including individuals with OCD in a ‘trigger’ disorder category, and future studies might potentially be better powered to compare the impact of COVID-19 between individuals with OCD and GAD.

## Conclusion

This longitudinal study examining the impact of the COVID-19 pandemic and its restrictions on individuals with pre-existing anxiety disorders at two time points six months apart demonstrated no significant overall change in symptomatology or functioning over time. Variability was, however, demonstrated between individuals, with some individuals describing ongoing anxiety, social isolation and concern for their future. Thus, despite COVID-19 having a minimal impact on many individuals with anxiety disorders; identifying those with ongoing symptoms or distress and providing multidisciplinary support to this cohort is suggested.
